# Gut commensal *Limosilactobacillus reuteri* induces atypical memory-like phenotype in human dendritic cells in vitro

**DOI:** 10.1080/19490976.2022.2045046

**Published:** 2022-03-08

**Authors:** Gintare Lasaviciute, Myriam Barz, Marieke van der Heiden, Claudia Arasa, Kanwal Tariq, Jaclyn Quin, Ann-Kristin Östlund Farrants, Eva Sverremark-Ekström

**Affiliations:** a Department of Molecular Biosciences, The Wenner-Gren Institute, Stockholm University, Stockholm, Sweden; b Central European Institute of Technology, Masaryk University, Brno, Czech Republic

**Keywords:** Limosilactobacillus reuteri, dendritic cells, T helper cells, innate immune memory, epigenetics

## Abstract

Memory-like responses in innate immune cells confer nonspecific protection against secondary exposures. A number of microbial agents have been found to induce enhanced or diminished recall responses in innate cells, however, studies investigating the ability of probiotic bacteria to trigger such effects are lacking. Here, we show that priming of human monocytes with a secretome from the gut probiotic bacterium *Limosilactobacillus* (*L*.) *reuteri* induces a mixed secondary response phenotype in monocyte-derived dendritic cells (mo-DCs), with a strong IL-6 and IL-1β response but low TNFα, IL-23 and IL-27 secretion. Instead, blood DC priming with *L. reuteri*-secretome resembles a tolerant state upon secondary exposure. A similar pattern was found in conventional and gut-like (retinoic acid exposed) DCs, although retinoic acid hampered TNFα and IL-6 production and enrichment of histone modifications in *L. reuteri*-secretome primed mo-DC cultures. Further, we show that the memory-like phenotype of mo-DCs, induced by priming stimuli, is important for subsequent T helper (Th) cell differentiation pathways and might determine the inflammatory nature of Th cells. We also show enhanced recall responses characterized by robust inflammatory cytokines and lactate production in the gut-like mo-DCs derived from β-glucan primed monocytes. Such responses were accompanied with enriched histone modifications at the promoter of genes associated with a trained phenotype in myeloid cells. Altogether, we demonstrate that a gut commensal-derived secretome prompts recall responses in human DCs which differ from that induced by classical training agents such as β-glucan. Our results could be beneficial for future therapeutic interventions where T cell responses are needed to be modulated.

## Graphical abstract

**Figure:**
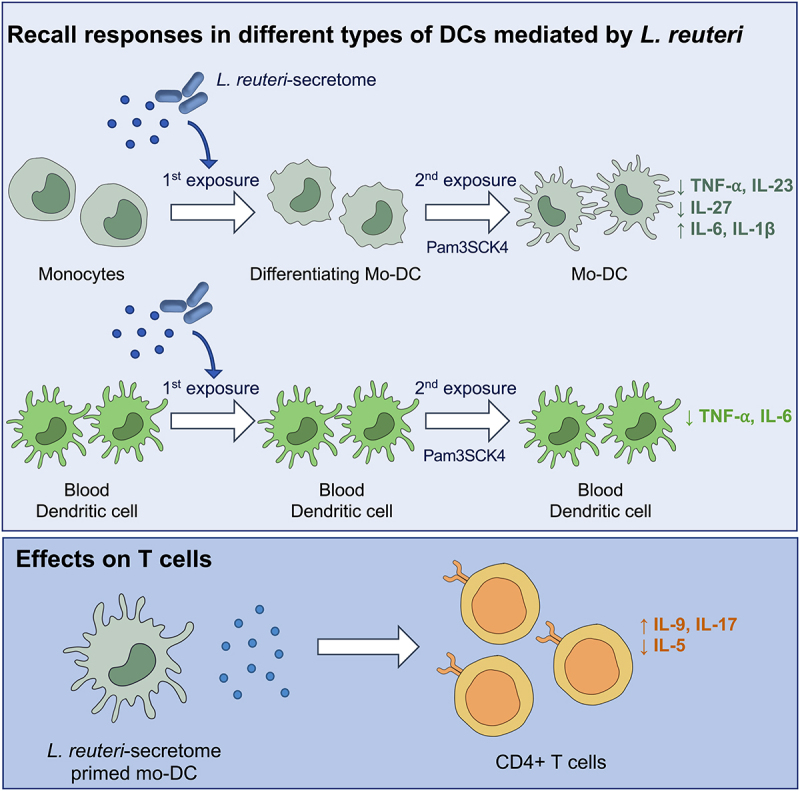

## Introduction

Over the last several years, there has been an immense research interest in how gut microbiota influence different aspects of host physiology. The gut microbiota regulate local immunity, but also intestinal bacteria-derived metabolites infiltrate different organs and shape host metabolism and immunological responses.^1–3^ It is well established that probiotic bacteria have prominent effects on adaptive and innate immune cell composition and function.^4–7^ However, little is known if innate cell exposure to probiotics can enhance their future reactivity to other microbial challenges which is nowadays termed as innate immune memory or training.^8,9^

Trained immunity is the increased host fitness upon vaccination or infection which is favorable during host defense. In contrast, tolerance helps to prevent excessive inflammatory responses which can be especially important for mucosal immunity in regards to colonizing microbes.^8,10^ The basis of innate memory lies in metabolic shifting and epigenetic reprogramming through histone modifications.^11^ To date, most studies have focused on innate memory induction in monocytes,^12^ macrophages^13^ or natural killer cells,^14^ while dendritic cells (DCs), which are the most efficient antigen-presenting cells capable of priming adaptive immune responses, have received much less attention.

Traditionally, DCs are classified into conventional DCs, plasmacytoid DCs and monocyte-derived DCs (mo-DCs).^15^ Although derived from different precursors, mo-DCs predominantly differentiate during inflammatory settings and produce DC-associated cytokines, including IL-23, IL-6, IL-1β and tumor necrosis factor (TNFα).^16^ The functional properties of DCs also depend on their resident location.^15^ For instance, a subset of intestinal DCs display the ability to synthesize retinoic acid (RA) which is a multifunctional metabolite of vitamin A.^15,17,18^ RA-producing DCs upregulate integrin α-chain CD103 and have a profound impact on mucosal homeostasis due to anti-inflammatory IL-10 production.^17,19^ Further, depending on the intestinal environment cues, DCs might redirect immune responses toward a pro- or anti-inflammatory profile through the initiation of T helper (Th) cell differentiation.

*Limosilactobacillus* (*L*.) *reuteri*, previously known as *Lactobacillus reuteri*, belongs to a group of gram-positive, lactic acid-producing bacteria. *L. reuteri* is capable of surviving throughout the whole gastrointestinal tract, does not carry transferable antibiotic resistant genes and easily adheres to the host epithelium, therefore, it is classified as competent probiotic bacteria.^20^ A great number of beneficial effects was shown to be induced by different *L. reuteri* strains, including protection against infections, increased absorption of vitamins, production of antimicrobial compounds, and modulation of immune responses.^6,20–22^

In the present study, we aimed to investigate if *L. reuteri*-derived metabolites imprint memory-like responses in distinct types of human DCs *in vitro* and if RA, which provides a gut-like DC phenotype,^17,19^ impacts their subsequent functional properties. Our results demonstrate that priming of monocytes or blood-derived DCs with the cell-free supernatant (CFS) from *L. reuteri* alters how these cells respond to future microbial re-stimulation, reflected both at DNA and protein levels. These characteristics are maintained following the addition of RA to the cultures, although the overall pro-inflammatory cytokine production in the gut-like DCs is alleviated. Importantly, we further show that priming events clearly influence how mo-DCs impact Th cell differentiation pathways. We also found that β-glucan priming of monocytes allows differentiated mo-DCs to elicit enhanced memory-like responses upon secondary challenge which reflects a trained innate cell-state, previously demonstrated in monocytes and macrophages. Since T cells govern the development of immune-mediated inflammatory diseases,^23^ de facto innate immune memory in DCs, which skew Th cell polarization, could be relevant for therapeutic strategies where excessive inflammatory responses are urged to be dampened or heightened.

## Results

### Short-term exposure to *L.* *reuteri-*CFS induces prominent mo-DC activation

**Figure 1.**
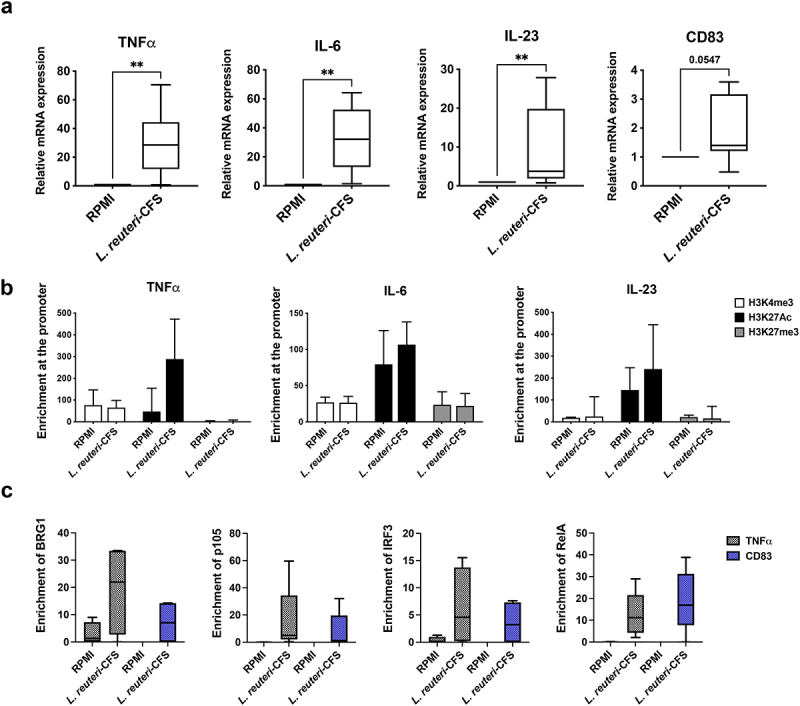
Conventional mo-DC short-term exposure to *L. reuteri*-CFS. Monocytes were differentiated to mo-DCs during 5 days culture without RA and then were stimulated with *L. reuteri*-CFS for 2 h or were left in culture medium RPMI as the control. (a) The expression of genes encoding TNFα, IL-6, IL-23 and CD83. Boxplots cover data between the 25th and the 75th percentile with median as the central line and whiskers showing min-to-max. Wilcoxon matched-pairs signed rank test was used to determine significant difference. (b) The enrichment of H3K4me3, H3K27Ac and H3K27me3 at the promoter of genes encoding TNFα, IL-6 and IL-23. The data are presented as median with interquartile range. (c) The enrichment of BRG1, p105, IRF3 and RelA at the promoters of genes encoding TNFα and CD83 genes. Boxplots cover data between the 25th and the 75th percentile with median as the central line and whiskers showing min-to-max, *p < .05, **p < .01, n = 4–9.

To test how the immunomodulatory probiotic bacterium *L. reuteri* influences mo-DC activation, we differentiated monocytes to mo-DCs, then exposed them to *L. reuteri*-CFS for 2 h and investigated DC-associated cytokines and maturation markers at the epigenetic and gene expression level. The expression of genes encoding TNFα, IL-6 and IL-23 was significantly upregulated upon *L. reuteri*-CFS exposure compared to the non-exposed cells (Figure 1a). The expression of gene encoding the maturation marker CD83 was also clearly enhanced following *L. reuteri*-CFS exposure (Figure 1a). Although non-significant, the level of histone H3 lysine 27 acetylation (H3K27Ac), which is associated with the active gene transcription, was higher at the promoters of all genes tested in *L. reuteri*-CFS exposed cells compared to the non-exposed cells (Figure 1b). Histone H3 lysine 4 trimethylation (H3K4me3) was comparable between the two groups, while the repressive mark histone H3 lysine 27 trimethylation (H3K27me3) was slightly lower in *L. reuteri*-CFS exposed cells (Figure 1b). In parallel, the enrichment of transcription factors BRG1, p105, IRF3 and RelA was also higher at the promoters of genes encoding DC-associated cytokines and surface markers compared to the control (Figure 1c, data only shown for TNFα and CD83).

### Monocyte priming with *L. reuteri*-CFS leads to atypical memory-like phenotype in mo-DCs upon secondary stimulus exposure

**Figure 2.**
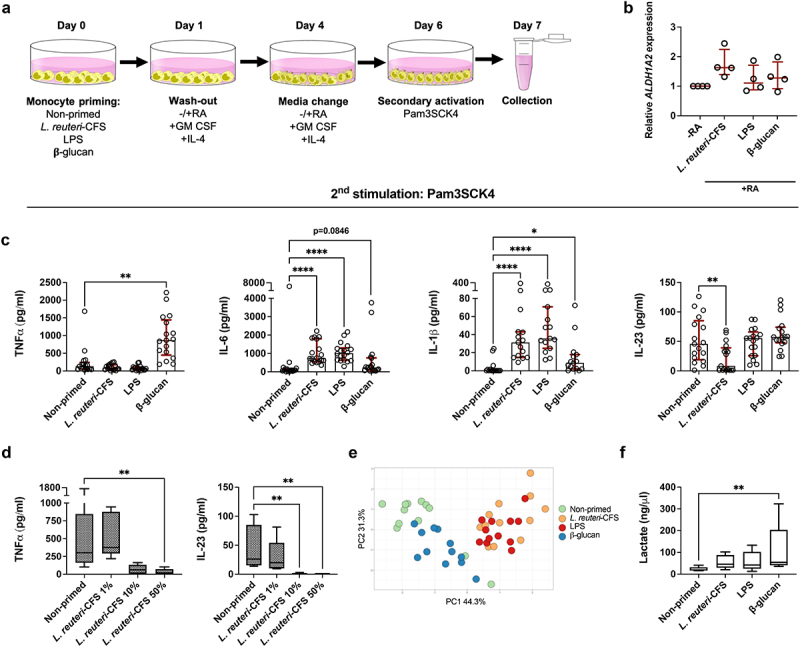
Recall responses in mo-DCs upon secondary stimulus exposure. Monocytes were primed with *L. reuteri*-CFS, LPS or β-glucan for 24 h, or were left non-primed as the control. Following wash-out of primary stimuli and differentiation to conventional mo-DCs (without RA) or gut-like mo-DCs (with RA), cells were exposed to Pam3SCK4 for 24 h. (a) *In vitro* experimental model of memory-like responses induction in mo-DCs. (b) The relative expression of *ALDH1A2* among conventional and gut-like mo-DCs. The data are presented as median with interquartile range. (c) The quantification of soluble TNFα, IL-6, IL-1β and IL-23 in the supernatant of gut-like mo-DCs. The data are presented as median with interquartile range. Paired Friedman test followed by Dunn’s multiple comparison was used to determine statistical difference between the control group and three priming conditions. (d) Soluble TNFα and IL-23 levels in the gut-like mo-DCs derived from monocytes primed with 1%, 10% or 50% of *L. reuteri*-CFS. Paired Friedman test followed by Dunn’s multiple comparison was used to determine statistical difference. (e) The PCA plot includes the log transformed data on the secretion of TNFα, IL-6, IL-1β and IL-23. The percentage of variance explained by the principle components (PC) 1 and 2 are indicated on the axes. (f) The secretion of L-lactate measured in the gut-like mo-DC cultures following lactate dehydrogenase removal. Boxplots cover data between the 25th and the 75th percentile with median as the central line and whiskers showing min-to-max. Paired Friedman test followed by Dunn’s multiple comparison was used to determine statistical difference, *p < .05, **p < .01, ***p < .001, ****p < .0001, n = 4–18.

To address the question whether *L. reuteri*-CFS imprints recall responses in mo-DCs, we primed monocytes with *L. reuteri*-CFS then washed the stimulus away and differentiated cells to conventional mo-DCs (without RA) or gut-like mo-DCs (with RA). Following differentiation, we exposed cells to the secondary stimulus triacylated lipopeptide Pam3CysSerLys4 (Pam3SCK4) (Figure 2a). As a control, we also primed monocytes with lipopolysaccharide (LPS) or β-glucan since they are known to induce diminished and enhanced recall responses in innate immune cells respectively^24^ (Figure 2a). Non-primed cells were exposed to culture medium alone and from now on are referred to as the control. A seven days interval between the first and the second exposures was chosen based on previous studies demonstrating that memory induction in innate cells takes up to 5–7 days in vitro.^25^ Priming with a first stimulus alters cells functional state which returns to the basal levels following stimulus removal. Therefore, a second exposure is introduced after a few days of incubation.

Gut-like characteristics of mo-DCs were confirmed by measuring the expression of the *ALDH1A2* gene which encodes the retinal aldehyde dehydrogenase (RALDH) enzyme required for RA synthesis.^17^ As expected, *ALDH1A2* expression was higher in the gut-like mo-DCs compared to conventional mo-DCs regardless of the primary stimuli (Figure 2b).

When monocytes were primed with *L. reuteri*-CFS or LPS, both gut-like- and conventional mo-DCs responded with low TNFα but elevated IL-6 and IL-1β response upon secondary stimulation, while IL-23 was significantly diminished only in *L. reuteri*-CFS primed cells compared to the control (Figure 2c for gut-like mo-DCs, Fig. S1 for conventional mo-DCs). An initial priming with β-glucan had no clear influence on the secondary responses in conventional mo-DCs (Fig. S1), whereas gut-like mo-DCs showed enhanced production of TNFα, IL-6 and IL-1β upon secondary stimulation, all in agreement with the trained innate cells phenotype (Figure 2c). Notably, the impact of *L. reuteri*-CFS on the TNFα and IL-23 secondary responses in conventional and gut-like mo-DCs was dose-dependent (Figure 2d for gut-like mo-DCs, Fig. S2 for conventional mo-DCs).

In addition, we measured the production of other DC-associated cytokines including TGF-β, IL-27 and IL-1RA. The secretion of TGF-β was comparable between the groups, while IL-27 was significantly diminished in *L. reuteri*-CFS primed mo-DC cultures regardless of RA addition (Fig. S3). Contrarily, IL-1RA was significantly enhanced only in β-glucan primed gut-like mo-DC cultures (Fig. S3).

The effects of RA were further evaluated by directly comparing cytokine secretion in conventional and gut-like mo-DCs, in donors where paired samples were available. In non-primed cultures, gut-like mo-DCs produced significantly lower levels of TNFα, IL-6 and IL-23 upon secondary stimulation (Fig. S4a). When monocytes were primed with *L. reuteri*-CFS or LPS, TNFα and IL-6 secretion were also significantly lower in the gut-like mo-DCs (Fig. S4b-c). In β-glucan primed cell cultures, IL-23 response was significantly lower in the gut-like mo-DCs (Fig. S4d).

Further, to emphasize the impact of primary stimuli on the functional phenotype of mo-DCs, we clustered gut-like mo-DC samples based on similarity of secreted soluble factors in a PCA plot. Gut-like mo-DCs from β-glucan primed monocytes clearly differed from the gut-like mo-DCs where monocytes were primed with LPS or *L. reuteri*-CFS, but also from mo-DCs derived from non-primed monocytes (Figure 2e).

Since trained innate cells have previously been shown to use glycolysis as a primary source of energy due to reduced oxygen consumption,^11^ we also measured the secretion of lactate – a final product of glycolysis – in the gut-like mo-DCs. Significantly enhanced lactate levels above those of the control were detected in the gut-like mo-DCs where monocytes had been primed with β-glucan (Figure 2f). In contrast, gut-like mo-DCs derived from monocytes primed with LPS or *L. reuteri*-CFS showed no obvious shift toward glycolysis as lactate levels in those cells were comparable to the control samples (Figure 2f).

### Recall cytokine responses correspond to chromatin changes in mo-DCs upon secondary activation

Epigenetic rewiring is the hallmark of innate memory induction,^26^ thus we next examined if the functional state of mo-DCs is associated with changes in H3K27Ac and H3K4me3 modifications at the promoters of DC-associated factors. Monocytes were primed with *L. reuteri*-CFS or β-glucan, or were left non-primed as the control. Following removal of the first stimuli, cells were differentiated to conventional mo-DCs or gut-like mo-DCs and were exposed to the secondary stimulus as described earlier.

**Figure 3.**
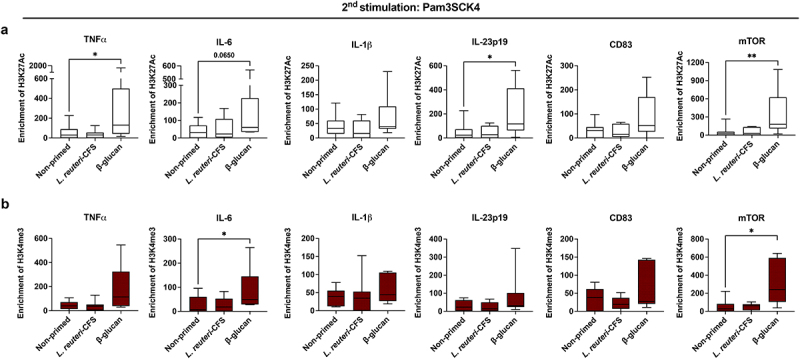
Epigenetic modifications in the gut-like mo-DCs upon secondary stimulation. Monocytes were primed with *L. reuteri*-CFS or β-glucan for 24 h, or were left non-primed as the control. Following wash-out of primary stimuli and differentiation to gut-like mo-DCs (with RA), cells were exposed to Pam3SCK4 for 24 h. (a) Boxplots show the enrichment of H3K27Ac and (b) the enrichment of H3K4me3 at the promoters of genes encoding TNFα, IL-6, IL-1β, IL-23, CD83 and mTOR. Boxplots cover data between the 25th and the 75th percentile with median as the central line and whiskers showing min-to-max. Paired Friedman test followed by Dunn’s multiple comparison was used to determine statistical difference between the control group and *L. reuteri*-CFS or β-glucan primed cell cultures, *p < .05, n = 7–8.

When monocytes were primed with *L. reuteri*-CFS, no significant differences of the H3K27Ac enrichment were found in the gut-like mo-DCs (Figure 3a) or conventional mo-DCs (Fig. S5a), compared to the control. H3K4me3 was somewhat enhanced at the promoter of IL-23 encoding gene in the *L. reuteri*-CFS primed conventional mo-DCs (Fig. S5b). Contrarily, when monocytes were primed with β-glucan, the level of H3K27Ac was increased at the promoters of TNFα, IL-6, IL-23 and mTOR encoding genes in the gut-like mo-DCs (Figure 3a), and at the promoters of TNFα and mTOR encoding genes in the conventional mo-DCs (Fig. S5a). H3K4me3 was significantly increased at the promoters of IL-6 and mTOR encoding genes in the gut-like mo-DCs (Figure 3b), and at the promoters of IL-23 and mTOR encoding genes in the conventional mo-DCs (Fig. S5b).

Further, we investigated the effects of RA on epigenetic modifications by comparing the enrichment of H3K27Ac and H3K4me3 at the promoters of genes in the conventional and gut-like mo-DCs where paired samples were available. When monocytes were primed with *L. reuteri*-CFS, the enrichment of H3K27Ac was markedly lower at the promoters of most genes in the gut-like mo-DCs compared to conventional mo-DCs (Fig. S6). In β-glucan primed cell cultures, no differences of histone enrichment were found between the two groups (data not shown).

### Blood DC priming with *L.reuteri*-CFS mediates diminished TNFα and IL-6 production upon secondary stimulus exposure

**Figure 4.**
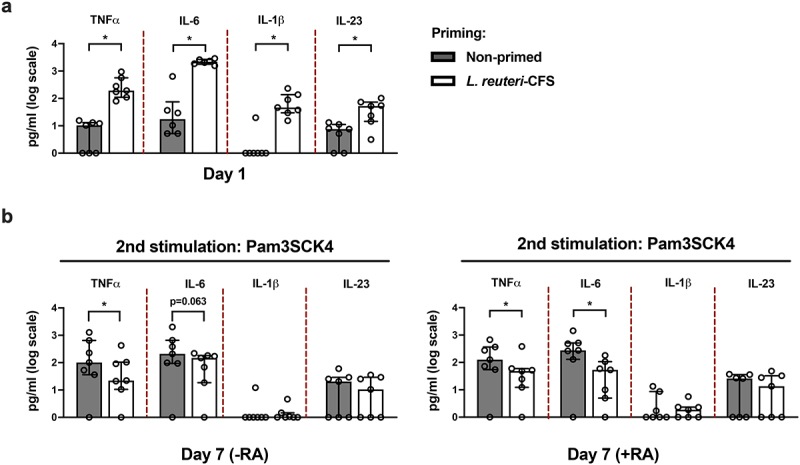
Blood DC priming with *L. reuteri*-CFS mediates diminished TNFα and IL-6 recall responses. Pan-DCs were primed with *L. reuteri*-CFS for 24 h or were left non-primed as the control. Following wash-out of primary stimulus and culture for 5 days with or without RA, Pam3SCK4 was added on day 6 for 24 h stimulation. (a) Soluble levels of TNFα, IL-6, IL-1β and IL-23 in DCs following 24 h priming with *L. reuteri*-CFS. (b) Soluble levels of TNFα, IL-6, IL-1β and IL-23 in the conventional DCs (-RA) and gut-like DCs (+RA) on day 7, upon secondary stimulus exposure. The data are presented as median with interquartile range, Wilcoxon matched-pairs signed rank test was used to determine statistical difference, *p < .05, n = 6–7.

We next investigated if primary blood DCs, which are not manipulated by additional cytokines during the resting phase, can also be imprinted by gut microbial factors to exhibit memory-like responses *in vitro* . Blood DCs were primed with *L. reuteri*-CFS for 24 h or were left non-primed as the control. Following wash-out of primary stimuli, we cultured DCs with or without RA and then exposed them to Pam3SCK4. The purity of blood DCs was evaluated by flow cytometry (Fig. S7). To examine the difference in activation upon primary and secondary stimuli, we collected the supernatants on day 1 (following 24 h priming) and day 7 (following 24 h of secondary stimulus). As expected, DCs were activated by *L. reuteri*-CFS at day 1 with secretion of all cytokines significantly enhanced compared to the control (Figure 4a). On day 7, TNFα and IL-6 were diminished in the conventional DCs (Figure 4b, left), and in the gut-like DCs (Figure 4b, right), derived from *L. reuteri*-CFS primed cells compared to the control.

### *L. reuteri*-CFS primed mo-DCs skew Th cell differentiation

**Figure 5.**
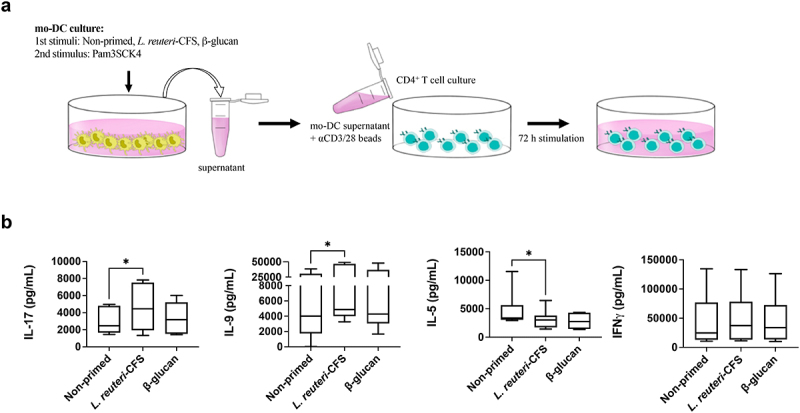
Mo-DCs differentiated from *L. reuteri*-CFS primed monocytes impact Th cell polarization. Monocytes were primed with *L. reuteri*-CFS or β-glucan for 24 h, or were left non-primed as the control. Following wash-out of primary stimuli and differentiation to gut-like mo-DCs, Pam3SCK4 was added for 24 h and supernatants were collected for Th cell polarization. The polarization was performed under T cell activating conditions. (a) *In vitro* experimental model of CD4 + T cell polarization. (b) The quantification of soluble IL-17, IL-9, IL-5 and IFN-γ in the Th cell cultures. Boxplots cover data between the 25th and the 75th percentile with median as the central line and whiskers showing min-to-max. Wilcoxon matched-pairs signed rank test was used to determine statistical difference, *p < .05, n = 7–10.

To study whether the mo-DCs, displaying memory-like responses, affect Th cell polarization, we cultured autologous Th cells with the supernatant from Pam3SCK4 challenged gut-like mo-DCs where monocytes had initially been primed with *L. reuteri*-CFS, β-glucan or were merely exposed to culture medium as the control (Figure 5a). The IL-17 and IL-9 responses were significantly enhanced compared to the control only in cultures where Th cells were exposed to supernatants from mo-DCs primed with *L. reuteri*-CFS (Figure 5b). Further, the production of IL-5 was significantly lower only in Th cells cultured with *L. reuteri*-CFS primed mo-DC supernatant, while no differences in IFN-γ secretion were detected between the groups (Figure 5b).

## Discussion

Despite of a variety of fungal, bacterial and viral agents which have been demonstrated to trigger protection against secondary exposures,^8^ little is known if probiotic bacteria can imprint innate immune cells to display enhanced or diminished recall responses. Lactobacilli are one of the most studied probiotics which modulate immune responses *in vivo* and *in vitro* .^6,20–22^ The exposure of mo-DCs to *L. reuteri*-CFS has previously been shown to induce IL-6, IL-23 and IL-10 secretion *in vitro* .^19^ In agreement, when we differentiated monocytes to conventional mo-DCs and then exposed them to *L. reuteri*-CFS for in short-term cultures, cells were clearly activated as the expression of genes encoding pro-inflammatory cytokines and maturation marker CD83 were upregulated. Blood DCs were also activated following priming with *L. reuteri*-CFS for 24 h in terms of pro-inflammatory cytokine production, however, most cytokines were diminished upon exposure to a secondary stimulus. Further, monocyte priming with the secretome from *L. reuteri* mediated low recall TNFα, IL-1RA, IL-23 and IL-27 production which all were dose-dependent, but strong IL-6 and IL-1β responses were found in both conventional and gut-like mo-DCs. Although we did not look at the surface expression of activation markers, collectively, these results support the notion that *L. reuteri*-CFS is able to promote substantial DC activation *in vitro,* but will at the same time induce a state that might be beneficial to future challenge. A recent study found that priming of human monocytes or murine bone marrow-derived macrophages (mBMMs) with live *L. plantarum* or *L. casei* bacteria prompts a tolerant memory-like state following secondary stimulus exposure.^27^ Tolerance induction was bacteria dose-dependent and it was not reciprocated when mBMMs were primed with *L. plantarum* conditioned medium.^27^ We cannot rule out the possibility that using live *L. reuteri* bacteria would lead to a different DC phenotype, however, studying bacteria-derived metabolites is equally relevant as they widely penetrate host tissues and small intestine is particularly prone to host-microbial metabolic exchange.^1^ Also, probiotic bacteria like lactobacilli have been shown to prompt the production of innate cell-associated cytokines through both cell-surface components and secreted metabolites.^28^

A number of components derived from *L. reuteri*-CFS could be potential candidates for the secondary responses induction in human DCs. We have previously shown that the CFS from *L. reuteri* DSM 17938 contains extracellular membrane vesicles which trigger monocytes activation comparable to *L. reuteri*-CFS stimulation alone.^29^ Others have shown that lactobacilli, including *L. reuteri*, secrete exopolysaccharides which can be recognized by DCs leading to distinct cytokine production.^30,31^ The cellular membrane of gram-positive bacteria such as lactobacilli comprises of lipoproteins, which as suggested by others, might be found in the conditioned media capable of activating Toll-like receptor (TLR) 2 in DCs leading to immunomodulatory effects.^32^ Nevertheless, a detailed investigation of specific *L. reuteri*-CFS components on secondary DC responses remains to be elucidated.

β-glucan is a cell wall component of fungi and bacteria which mediates innate cell training through dectin-1 receptor recognition. Its activation leads to the signaling of mammalian target of rapamycin (mTOR) pathway, metabolic and epigenetic rewiring, and increased pro-inflammatory cytokine production upon secondary stimulation.^24,33,34^ Indeed, we found enhanced recall TNFα, IL-1β, IL-6, IL-1RA and lactate production in the gut-like mo-DCs derived from β-glucan primed monocytes. Increased lactate production is a hallmark of glycolysis commitment. Trained innate cells preferentially undergo metabolic transition toward glycolysis in order to prepare for a more rapid but sufficient source of energy which is required to confer nonspecific protection against stimuli re-exposure measured as a higher cytokine response.^11,35^ Genome-wide histone modification profiling 7 days post β-glucan training of monocytes has previously revealed that active histone marks H3K27Ac and H3K4me3 are enriched in genes linked to glycolysis and metabolism, such as mTOR.^11^ These two histone modifications have also been reported to be enriched at the promoter of genes encoding pro-inflammatory cytokines, including TNFα and IL-6, which are primarily associated with a trained innate cell state.^36^ Our data are in line with the former findings since the enrichment of both H3K27Ac and H3K4me3 was markedly higher at the promoters of distinct genes in mo-DCs derived from β-glucan primed monocytes. Hence, we conclude that monocytes can be educated to display enhanced cytokine recall responses even after differentiation to mo-DCs, and that epigenetic modifications after initial monocyte stimulation play a vital role during such process.

Contrarily to β-glucan, a high dose of LPS is a classical agent for tolerance induction in innate cell populations.^8,24^ In agreement, blood DC-priming with LPS clearly induced DCtolerance as all measured cytokines were completely abolished upon secondary stimulus exposure (data not shown). LPS priming of monocytes induced similar memory-like responses in mo-DCs as did *L. reuteri*-CFS, a robust IL-6 and IL-1β production but low TNFα secretion. This indicates that DCs respond differently to primary stimuli, depending on the environment and which precursors they are derived from.

RA is an integral component of intestinal immunity. It is generated by RALDH enzyme which is expressed in various cell types including DCs.^18^ It has previously been shown that *L. reuteri*-CFS stimulates TLR2 signaling which in turn induces RALDH expression.^19^ The same study suggested that the upregulation of TLR2 could be a positive feedback mechanism induced by *L. reuteri*-CFS to potentiate RA effects on mo-DCs.^19^ Indeed, we found that gut-like mo-DCs derived from *L. reuteri*-CFS-primed monocytes expressed higher levels of *ALDH1A2* gene encoding RALDH compared to the conventional mo-DCs. Also, while pro-inflammatory cytokines were diminished in all mo-DC cultures differentiated in the presence of RA, the enrichment of active histonemodifications were only reduced at the promoters of genes in the gut-like mo-DCs derived from *L. reuteri*-CFS-primed monocytes. Interestingly, we further found that enhanced secondary responses in mo-DCs induced by β-glucan priming of monocytes are not influenced by RA, although it has previously been shown to downregulate trained immunity in monocyte cultures *in vitro*.^37^ The main reason for the discrepancies between the two studies could be the design of experimental model. We added RA during mo-DC differentiation after priming, while in the latter study monocytes were exposed to RA together with the primary training stimulus,^37^ which may interfere with the re-programming of monocytes.

In the gut, DCs are responsible not only for combating local pathogens, but also for subsequent Th cell polarization and immune regulation.^38^ Due to limited numbers of blood DCs, we cultured autologous Th cells with the supernatant from gut-like mo-DCs primed with β-glucan or *L. reuteri*-CFS. Regardless of the priming agent, we found a great production of IL-17 which is the main cytokine secreted by Th17 cells.^39^ The dichotomous nature of Th17 cells depends on the IL-23 levels.^40^ For instance, Th cells cultured with IL-6 and TGF-β are polarized to a nonpathogenic Th17 cell subtype important for host defense, whereas Th cells cultured in the presence of IL-6, IL-1β and IL-23 develop into inflammatory Th17 cells.^40^ As IL-23 was abolished only in *L. reuteri*-CFS primed mo-DC cultures, we speculate that *L. reuteri*-CFS induces non-inflammatory Th17 cell differentiation by modulating mo-DC function *in vitro*. This is in contrast to Th cells which had been cultured with the supernatant from β-glucan primed mo-DCs containing abundant levels of IL-23. Further, we found enhanced IL-9 secretion, while IL-5 levels were alleviated in the Th cells which had been cultured with the supernatant from *L. reuteri*-CFS primed mo-DC cultures. The cytokine IL-5 is associated with Th2 cell responses, while IL-9 is the main product of the Th9 cells, although if Th2 and Th9 cells really represent separate lineages is not yet clear.^41^ Both aforementioned cytokines are implicated in allergic disease^41,42^ or atopic dermatitis development.^43^ Gut commensals like different lactobacilli have been associated with lower risk of developing allergic disease and a reduced Th2-associated immune profile.^44^ The concomitant and perhaps surprising increase in IL-9 could be a result of the significant reduction of mo-DC-produced IL-27 following *L. reuteri*-CFS priming, as IL-27 is a potent inhibitor of Th9 cell differentiation.^45^ The continuous exposure to various microbes in the gut environment, and their ability to skew Th responses in a way that could have consequences also for peripheral immunity, illustrate the importance of an environment that can balance and tune signals given to T cells in this compartment.

We acknowledge that using live *L. reuteri* bacteria in parallel to *L. reuteri*-CFS could have contributed additional information to our study. *L. reuteri* contains a number of cell surface components, such as lipoteichoic acid, which exhibit immunomodulatory effects,^46^ hence whole bacteria often provoke different immune cell outcome. Alternatively, *in vivo* experiments would better reflect our approach to use *L. reuteri*-CFS since the contact between immune cells and microbial populations in the gut is limited,^47^ while microbial metabolites are able to penetrate host tissues and instigate cellular responses.^1^ Of note, the effects of lactobacilli *in vivo* greatly vary depending on the bacterial strain and organism. For instance, oral administration with *L. reuteri* CCFM1132, but not *L. reuteri* CCFM1040, has been shown to reduce the production of IL-23/Th17 axis-associated cytokines in a murine psoriasis-like model.^48^ Newborn rats fed with formula supplemented with *L. reuteri* DSM 17938, but not *L. reuteri* ATCC PTA 4659, showed diminished intestinal TNFα, IL-1β and IFN-γ production compared to the rats fed with formula alone.^49^ Another study has shown that oral gavage with *L. reuteri* ATCC PTA 4659 significantly diminished colonic IL-1β and IL-6, but not TNFα, production in a murine colitis model.^50^ Therefore, *in vivo* studies using different *L. reuteri* strains would also help to uncover whether memory-like responses in innate cells are strain-specific.

It has recently been reported that *Cryptococcus neoformans* induce murine splenic DCtraining following re-stimulation with the same pathogen.^51^ To the best of our knowledge, here we are the first to demonstrate that priming of human blood DCs with the CFS from the probiotic gut bacterium *L. reuteri* induces tolerant recall responses upon secondary exposure. An initial activation of monocytes with *L. reuteri*-CFS generates mixed recall responses phenotype in mo-DCs which differs from that induced by classical training or tolerance agents, such as β-glucan. We further show that *L. reuteri*-CFS-priming of monocytes before cell differentiation to mo-DCs strongly impacts subsequent T cell responses. Signals derived from the microbiome, like bacteria and their metabolites, divert the immune cells toward pro- or anti-inflammatory responses, which often shape the host’s susceptibility to diseases.^52^ Further, microbial metabolites, including those produced by probiotics, have the potential to travel to the bone marrow where they epigenetically imprint hematopoietic progenitors.^53,54^ Therefore, selective modulation of the intestinal microbiota could have long-term benefits to the host and it is of great importance to determine which probiotics or probiotic compounds that are capable of imprinting memory responses in innate cells. This could be beneficial for future therapeutic interventions where, for instance, excessive inflammation is necessary to be regulated.

## Materials and methods

### Human subjects

Buffy coats were obtained as rest products from anonymous, healthy individuals donating blood. The biological material cannot be traced back to any individual and the project does therefore not require approval from the Swedish ethical review authority.

### Monocytes and CD4+ Th cell enrichment from peripheral blood mononuclear cells

Peripheral blood mononuclear cells (PBMCs) were isolated from buffy coats using Ficoll-Hypaque (Cytiva) gradient centrifugation. Monocytes and Th cells were further separated from PBMCs by negative selection using EasySep^TM^ human monocyte and CD4 + T cell enrichment kit respectively (both from STEMCELL Technologies) according to the manufacturer’s instructions.

### Monocyte differentiation to dendritic cells and innate memory induction

Isolated monocytes were seeded at 1 × 10^6^ cells/ml in 24-well plates (with 400 μl/well) or 6-well plates (with 2 ml/well) using complete culture medium containing RPMI-1640 supplemented with 20 mM HEPES, 2 mM L-glutamine, 100 U/ml penicillin, 100 μg/ml streptomycin (all from Cytiva), 5% human serum (Sigma-Aldrich), 50 μM 2-mercaptoethanol and 2% sodium pyruvate (both from Gibco). Following the adherence for 1–2 h, monocytes were primed with 100 μg/ml whole β-glucan particles from *Saccharomyces cerevisiae* (InvivoGen), 100 ng/ml LPS from *Escherichia coli* 055:B5 (Sigma-Aldrich), 10% *L. reuteri*-CFS or were kept in RPMI as the control. For some experiments, monocytes were also exposed to 1% or 50% of *L. reuteri*-CFS. Following 24 h incubation, cells were washed twice with warm phosphate-buffered saline (PBS, Sigma-Aldrich) and complete culture medium containing 35 ng/ml recombinant human (rh) IL-4 and 50 ng/ml rh GM-CSF (both from PeproTech) was added. For some experiments, all-trans-RA (Sigma-Aldrich) was added at a final concentration of 1,15 μg/ml. On day 4, half of the medium volume was replaced with the fresh medium containing rh IL-4, rh GM-CSF and RA when required. On day 6, cells were exposed to the second stimulation with 10 μg/ml of Pam3SCK4 (InvivoGen) or were kept in RPMI. Following 24 h incubation, supernatants were collected and stored at −20°C until further analysis while cells were collected for chromatin immunoprecipitation (ChIP) and qPCR. Throughout the experiment, cells were incubated at 37°C with 5% CO_2_.

### Pan-DC enrichment and stimulation

PBMCs were isolated from buffy coats as described earlier and pan-DCs (including myeloid and plasmacytoid DCs) were further enriched by negative selection using EasySep^TM^ human pan-DC pre-enrichment kit (STEMCELL Technologies) according to the manufacturer´s instructions. Cell purity of enriched live (fixable viability stain 780 ^–^ from BD Biosciences), lineage negative cells (CD14^–^ (clone M5E2, BD Biosciences), CD16^–^ (clone 3G8, BD Biosciences), CD3^–^ (clone UCHT1 BD Biosciences), CD20^–^ (clone 2H7, Biolegend) or CD19^–^ (clone HIB19, Biolegend), CD56^–^ (clone MEM-188, Biolegend), but HLA DR^+^ (clone L243, Biolegend)) was confirmed by flow cytometry. Isolated DCs were seeded at 1 × 10^6^ cells/ml in 96-well plates with 200 μl/well complete culture medium. Following 1–2 h rest, DCs were primed with 10% *L. reuteri*-CFS or were kept in RPMI as the control. Following 24 h incubation, cells were collected and washed twice with warm PBS and fresh medium with RA (1,15 μg/ml) or without RA was added. On day 4, the medium was replaced and on day 6 cells were re-activated with 10 μg/ml of Pam3SCK4 for 24 h. Supernatants were collected following 24 h priming with the first stimuli on day 1 and following 24 h stimulation with the second stimulus on day 7. Supernatants were stored at −20°C until further analysis.

### *Short-term stimulation of monocyte-derived dendritic cells with* L. reuteri*-CFS*

Isolated monocytes were seeded at 1 × 10^6^ cells/ml in 6-well plates (with 2 ml/well) complete culture medium containing 35 ng/ml rh IL-4 and 50 ng/ml rh GM-CSF. On day 3, the medium was replenished and on day 5 cells were stimulated with 10% *L. reuteri*-CFS or were kept in RPMI as the control. Following 2 h incubation, cells were collected for ChIP or mRNA expression by qPCR analysis.

### CD4 + T cell culture experiments

A total of 0.1 × 10^6^ enriched autologous Th cells were seeded in 96-well plates with RPMI culture medium supplemented with 20 mM HEPES, 2 mM L-glutamine, 100 U/ml penicillin, 100 μg/ml streptomycin and 10% heat-inactivated fetal bovine serum (FBS, Sigma Aldrich). The supernatant from gut-like mo-DCs (derived from monocytes primed with β-glucan, *L. reuteri*-CFS or non-primed monocytes) was thawed and added to the Th cell cultures (constituted a total of 80% of T cell culture medium, total 200 μl/well). The human T-activator CD3/CD28 Dynabeads (Gibco) were added at a 2:1 cell-to-bead ratio at the same time to activate T cells. Cells were incubated for 72 h at 37°C with 5% CO_2_ and the supernatant was collected for downstream applications.

### *Generation of cell-free supernatant from* L. reuteri

*L. reuteri* DSM 17938, a kind gift from BioGaia AB (Stockholm Sweden) was grown on Rogosa agar plates for 24 h. A single colony was then inoculated into De Man, Rogosa and Sharpe (MRS) medium and grown as still culture overnight. The bacteria were centrifugated to separate the pellet and re-suspended in RPMI-1640 medium supplemented with 18 g/l glucose and 20% FBS at OD of 0.2 and grown as still cultures for 48 h at 37°C, 5% CO_2_. The bacteria were pelleted by centrifugation once again and 5 M NaOH was used to neutralize the pH. The CFS was collected, 0.2 μm filtered and stored at −20°C.

### Chromatin immunoprecipitation

ChIP analyses were performed as described elsewhere.^55^ Briefly, chromatin from different treatment groups was cross-linked using formaldehyde (Sigma-Aldrich). Diagenode Biorupter was used to sonicate cell preparations for 15 min × 3 (30 s on; 30 s off). Chromatin from 1 × 10^6^ cells was incubated with 1 μg of IgG (ab171870), H3 (ab1791), H3K27Ac (ab4729), H3K27me3 (ab6002), H3K4me3 (ab8580), RelA (NFκB p65, ab7970), NFκB p105 (p50, ab7971), IRF3 (ab76409), (all from Abcam), or BRG1 antibody (generated as described elsewhere^56^) overnight at 4°C. Protein A/G magnetic beads (ThermoScientific^TM^) were incubated with chromatin/antibody mix for 1 h at 4°C. Phenol/chloroform separation was used to purify DNA. The precipitated DNA fragments were amplified by qPCR using KAPA SYBR FAST qPCR Master mix (Kappa Biosystems Inc.) and Corbett Research RG-6000 real-time PCR Cycler (Corbett Research). The results are presented as percentage of input, with the IgG control subtracted from the Ct values obtained in the samples precipitated with specific antibodies. Primer pair list can be found in the Table S1. Primers were purchased from Eurofins or Qiagen.

### RNA extraction, cDNA synthesis and quantitative PCR

QuickRNA MiniPrep kit (Zymo Research) was used to extract total RNA and SuperScript VILO cDNA synthesis kit (Invitrogen) was used for reverse transcription of RNA according to the manufacturer’s instructions. qPCR was performed using KAPA SYBR FAST qPCR Master mix and Corbett Research RG-6000 real-time PCR Cycler. mRNA expression levels were calculated using 2^−ΔΔCt^ method and PP1A was used for normalization. Primer pair list can be found in Table S1.

### Enzyme-linked immunosorbent assay

The soluble levels of TNFα, IL-6, IL-1β, IL-23, TGF-β, IL-27, IL-17, IL-5, IFN-γ (all from MabTech AB) and IL-9, IL-RA (both from R&D systems, Bio-Techne) were measured in the cell culture supernatant according to the instructions from the manufacturer. The results were analyzed using SoftMax Pro 5.2 rev C (Molecular Devices Corp.).

### L-Lactate measurement

The cell culture supernatant was centrifuged at 12,000 g for 30 min and filtered using 10 kDa Amicon Ultra-0.5 Centrifugal Filter Units (Millipore) to remove lactate dehydrogenase. L-Lactate was measured using Lactate assay kit II (Sigma-Aldrich) according to the instructions from the manufacturer.

### Statistical analysis

GraphPad Prism 8 (GraphPad Prism Inc.) was used for the statistical analysis. Paired Friedman test followed by Dunn’s multiple comparison or Wilcoxon matched-pairs signed rank test was applied to determine the differences between distinct condition groups. P-values <0.05 were considered statistically significant. All plots show the median with interquartile range, n = number of donors. All of the statistical details of experiments can be found in the figure legends.

The principle component analyses (PCA) were performed in RStudio 1.3.5033 (RStudio Inc.). The data were divided into two principle components, of which the amount of variance in the data that is explained by the component is mentioned as a percentage on the axes. The parameters used in the PCA are mentioned in the figure legends.

## Supplementary Material

Supplemental Material

## Data Availability

The authors confirm that the data supporting the findings of this study are available within the article and its supplementary materials.

## Supplementary material

Supplementary material can be assessed here.
