# Supplementary material for: Gut commensal Limosilactobacillus reuteri induces atypical memory-like phenotype in human dendritic cells in vitro
Source: Gut Microbes. 2022 Mar 8;14(1):2045046. doi: 10.1080/19490976.2022.2045046 (PMC8920211; doi:10.1080/19490976.2022.2045046)
Supplement: Supplemental Material [file KGMI_A_2045046_SM0954.docx]

**Supplementary Materials**

Supplementary file 1. Secondary responses in conventional mo-DCs.

Supplementary file 2. Secondary TNF$\alpha$ and IL-23 responses in conventional mo-DCs depend on a dose of priming stimulus *L. reuteri*-CFS.

Supplementary file 3. Secondary TGFβ, IL-27 and IL-1RA responses in mo-DCs.

Supplementary file 4. The comparison of secondary responses between conventional and gut-like mo-DCs.

Supplementary file 5. Epigenetic modifications in conventional mo-DCs upon secondary stimulus exposure.

Supplementary file 6. The comparison of epigenetic modifications between conventional and gut-like mo-DCs derived from *L. reuteri*-CFS primed monocytes.

Supplementary file 7. The representative gating strategy for pan-DCs.

**Supplementary file 1. Secondary responses in conventional mo-DCs.** Monocytes were primed with *L. reuteri*-CFS, LPS or β-glucan for 24 h, or were left non-primed as the control. Following wash-out of primary stimuli and differentiation to conventional mo-DCs (without RA), cells were exposed to Pam3SCK4 for 24 h. The graphs show soluble TNF$\alpha$, IL-6, IL-1β and IL-23 levels in the culture supernatant. The data are presented as median with interquartile range. Paired Friedman test followed by Dunn´s multiple comparison was used to determine statistical difference between the control group and three priming conditions, *p <0.05, **p <0.01, ***p <0.001, ****p <0.0001, n=9-11.

**Supplementary file 2. Secondary TNF**$\boldsymbol{\alpha}$ **and IL-23 responses in conventional mo-DCs depend on a dose of priming stimulus *L. reuteri*-CFS.** Monocytes were primed with different concentration of *L. reuteri*-CFS for 24 h, or were left non-primed as the control. Following wash-out of primary stimuli and differentiation to conventional mo-DCs (without RA), cells were exposed to Pam3SCK4 for 24 h. Boxplots show soluble TNF$\alpha$ and IL-23 levels in the conventional mo-DCs derived from monocytes primed with 1%, 10% or 50% of *L. reuteri*-CFS. Paired Friedman test followed by Dunn´s multiple comparison was used to determine statistical difference, **p <0.01, n=6.

**Supplementary file 3. Recall TGFβ, IL-27 and IL-1RA responses in mo-DCs.** Monocytes were primed with *L. reuteri*-CFS, LPS or β-glucan for 24 h, or were left non-primed as the control. Following wash-out of primary stimuli and differentiation to conventional mo-DCs (without RA) or gut-like mo-DCs (with RA), cells were exposed to Pam3SCK4 for 24 h. The graphs show soluble TGFβ, IL-27 and IL-1RA levels in the culture supernatant. The data are presented as median with interquartile range. Paired Friedman test followed by Dunn´s multiple comparison was used to determine statistical difference between the control group and three priming conditions, *p <0.05, **p <0.01, n=2-11.

**Supplementary file 4.** **The comparison of secondary cytokine responses between conventional and gut-like mo-DCs.** Monocytes were primed with *L. reuteri*-CFS, LPS or β-glucan for 24 h, or were left non-primed as the control. Following wash-out of primary stimuli and differentiation to conventional mo-DCs (without RA) or gut-like mo-DCs (with RA), cells were exposed to Pam3SCK4 for 24 h. **(a)** The quantification of soluble TNF$\alpha$, IL-6, IL-1β and IL-23 in mo-DCs derived from non-primed monocytes, **(b)** *L. reuteri*-CFS, **(c)** LPS or **(d)** β-glucan, primed monocytes. Wilcoxon matched-pairs signed rank test was used to determine significant difference. *p <0.05, **p <0.01, ***p <0.001, n=9-11.

**Supplementary file 5. Epigenetic modifications in conventional mo-DCs upon secondary stimulus exposure.** Monocytes were primed with *L. reuteri*-CFS or β-glucan for 24 h, or were left non-primed as the control. Following wash-out of primary stimuli and differentiation to conventional mo-DCs (without RA), cells were exposed to Pam3SCK4 for 24 h. **(a-b)** The enrichment of H3K27Ac (a) or H3K4me3 (b) at the promoters of genes encoding TNF$\alpha$, IL-6, IL-1β, IL-23, CD83 and mTOR. Boxplots cover data between the 25th and the 75th percentile with median as the central line and whiskers showing min-to-max. Paired Friedman test followed by Dunn´s multiple comparison was used to determine statistical difference between the control group and *L. reuteri*-CFS or β-glucan primed cell cultures, *p <0.05, **p <0.01, n=6-7.

**Supplementary file 6. The comparison of epigenetic modifications between conventional and gut-like mo-DCs derived from *L. reuteri*-CFS primed monocytes.** Monocytes were primed with *L. reuteri*-CFS for 24 h or were left non-primed as the control. Following wash-out of primary stimulus and differentiation to conventional mo-DCs (without RA) or gut-like mo-DCs (with RA), cells were exposed to Pam3SCK4 for 24 h. Graphs show the enrichment of H3K27Ac or H3K4me3 at the promoters of genes encoding TNF$\alpha$, IL-6, IL-1β, IL-23, CD83 and mTOR. Wilcoxon matched-pairs signed rank test was used to determine significant difference, *p <0.05, n=5-6.

**Supplementary file 7. The representative gating strategy for pan-DCs.** The purity of enriched blood-derived pan-DCs was determined by flow cytometry gating single, live, lineage negative (CD14^–^CD16^–^CD3^–^CD56^–^CD20^–^or CD19^–^) but HLA DR^+^ cells.

### Table S1. Primer pair sequences.

| **Gene**  **qPCR experiments** | **Forward (F) / Reverse (R)** | **Oligonucleotides** | **References** |
| --- | --- | --- | --- |
| DC SIGN | F | TTG TTG GGC TCT CCT CTG TT | (1) |
|  | R | AAG TAA CCG CTT CAC CTG GA |  |
| IL-6 | F | TAG AGC TTC TCT TTC GTT CCC GGT | (2) |
|  | R | TGT GTC TTG CGA TGC TAA AGG ACG |  |
| IL-23 | F | CTC TGC TCC CTG ATA GCC CT | (3) |
|  | R | TGC GAA GGA TTT TGA AGC GG |  |
| IL-10 | F | GCC TAA CAT GCT TCG AGA TC | (4) |
|  | R | CTC ATG GCT TTG TAG ATG CC |  |
| CD83 | F | ATT CCC TGA AGA TCC GAA AC | (5) |
|  | R | GAA AAT AAC CAG AGC CAG CA |  |
| CD86 | F | GTT GCC TTG AGC AAA AAC AA | (5) |
|  | R | TGA GAG AGG AAG AGC TGC AA |  |
| ALDH1A2 | F | TTG GTT CAG TGT GGA GAA GG | (6) |
|  | R | AAA GCT TGC AGG AAT GGT TTG |  |
| PP1A | F | AGA CAA GGT CCC AAA GAC | (7) |
|  | R | ACC ACC CTG ACA CAT AAA |  |
| **ChIP experiments** | **Forward (F) / Reverse (R)** | **Oligonucleotides** | **References** |
| TNF promoter | F | CAGGCAGGTTCTCTTCCTCT | (8) |
|  | R | GCTTTCAGTGCTCATGGTGT |  |
| IL-1β promoter | F | CACTCTTCCACTCCCTCC | (9) |
|  | R | AGCCTCAAACCCTTCCTC |  |
| IL-6 promoter | F | TAGCCTCAATGACGACCTAAG | (10) |
|  | R | GTGGGGCTGATTGGAAACCT |  |
| IL-23p19 promoter | F | GGCCTCATTCTGACGTCTTC | (11) |
|  | R | CTGAAGGACCAGCCAGAGTC |  |
| DC SIGN promoter | F | ATCACAGGGTGGGAAATAA | (12) |
|  | R | AGTCTTGGTTCCTTGGAGTC |  |
| CD86 promoter | F | GCTCATCTTAACGTCATGTCTG | Qiagen |
|  | R | ATTTAACCCTTTCCTTGCAGTT |  |
| CD83 promoter | F | ACATTGGTGTCGAGTTGGAG | Qiagen |
|  | R | GGTCTT CCTGGGGTGTCTC |  |
| mTOR promoter | F | ATAAAGAGCGCTAGCCCGAA | (13) |
|  | R | GGTCTTCCTGGGGTGTCTC |  |
| IL-10 promoter | F | CTCCCCAGGAAATCAACT | (14) |
|  | R | AAAAGCCACAATCAAGGT |  |

**References**

1. Lech M, Susanti HE, Römmele C, Gröbmayr R, Günthner R, Anders HJ. Quantitative expression of C-Type lectin receptors in humans and mice. *Int J Mol Sci* (2012) **13**:10113–10131. doi:10.3390/ijms130810113

2. Ghosh CC, Ramaswami S, Juvekar A, Vu H-Y, Galdieri L, Davidson D, Vancurova I. Gene-Specific Repression of Proinflammatory Cytokines in Stimulated Human Macrophages by Nuclear IκBα. *J Immunol* (2010) **185**:3685–3693. doi:10.4049/jimmunol.0902230

3. Shi Q, Yin Z, Zhao B, Sun F, Yu H, Yin X, Zhang L, Wang S. PGE2 Elevates IL-23 Production in Human Dendritic Cells via a cAMP Dependent Pathway. *Mediators Inflamm* (2015) **2015**: doi:10.1155/2015/984690

4. Staples KJ, Smallie T, Williams LM, Foey A, Burke B, Foxwell BMJ, Ziegler-Heitbrock L. IL-10 Induces IL-10 in Primary Human Monocyte-Derived Macrophages via the Transcription Factor Stat3. *J Immunol* (2007) **178**:4779–4785. doi:10.4049/jimmunol.178.8.4779

5. Bujila I, Schwarzer E, Skorokhod O, Weidner JM, Troye-Blomberg M, Östlund Farrants AK. Malaria-derived hemozoin exerts early modulatory effects on the phenotype and maturation of human dendritic cells. *Cell Microbiol* (2016) **18**:413–423. doi:10.1111/cmi.12521

6. Kim EW, De Leon A, Jiang Z, Radu RA, Martineau AR, Chan ED, Bai X, Su W-L, Montoya DJ, Modlin RL, et al. Vitamin A Metabolism by Dendritic Cells Triggers an Antimicrobial Response against Mycobacterium tuberculosis . *mSphere* (2019) **4**:1–14. doi:10.1128/msphere.00327-19

7. Quin JE, Bujila I, Chérif M, Sanou GS, Qu Y, Homann MV, Rolicka A, Sirima SB, O’Connell MA, Lennartsson A, et al. Major transcriptional changes observed in the Fulani, an ethnic group less susceptible to malaria. *Elife* (2017) **6**:1–19. doi:10.7554/eLife.29156

8. Kleinnijenhuis J, Quintin J, Preijers F, Joosten LAB, Ifrim DC, Saeed S, Jacobs C, Van Loenhout J, De Jong D, Hendrik S, et al. Bacille Calmette-Guérin induces NOD2-dependent nonspecific protection from reinfection via epigenetic reprogramming of monocytes. *Proc Natl Acad Sci U S A* (2012) **109**:17537–17542. doi:10.1073/pnas.1202870109

9. Chan C, Li L, McCall CE, Yoza BK. Endotoxin Tolerance Disrupts Chromatin Remodeling and NF-κB Transactivation at the IL-1β Promoter. *J Immunol* (2005) **175**:461–468. doi:10.4049/jimmunol.175.1.461

10. Zimmermann M, Aguilera FB, Castellucci M, Rossato M, Costa S, Lunardi C, Ostuni R, Girolomoni G, Natoli G, Bazzoni F, et al. Chromatin remodelling and autocrine TNFα are required for optimal interleukin-6 expression in activated human neutrophils. *Nat Commun* (2015) **6**: doi:10.1038/ncomms7061

11. Goodall JC, Wu C, Zhang Y, McNeill L, Ellis L, Saudek V, Gaston JSH. Endoplasmic reticulum stress-induced transcription factor, CHOP, is crucial for dendritic cell IL-23 expression. *Proc Natl Acad Sci U S A* (2010) **107**:17698–17703. doi:10.1073/pnas.1011736107

12. Bullwinkel J, Lüdemann A, Debarry J, Singh PB. Epigenotype switching at the CD14 and CD209 genes during differentiation of human monocytes to dendritic cells. *Epigenetics* (2011) **6**:45–51. doi:10.4161/epi.6.1.13314

13. Schrum JE, Crabtree JN, Dobbs KR, Kiritsy MC, Reed GW, Gazzinelli RT, Netea MG, Kazura JW, Dent AE, Fitzgerald KA, et al. Plasmodium falciparum Induces Trained Innate Immunity. *J Immunol* (2018) **200**:1243–1248. doi:10.4049/jimmunol.1701010

14. Fu LH, Ma CL, Cong B, Li SJ, Chen HY, Zhang JG. Hypomethylation of proximal CpG motif of interleukin-10 promoter regulates its expression in human rheumatoid arthritis. *Acta Pharmacol Sin* (2011) **32**:1373–1380. doi:10.1038/aps.2011.98
